# Stomach fullness shapes prey choice decisions in crab plovers (*Dromas ardeola*)

**DOI:** 10.1371/journal.pone.0194824

**Published:** 2018-04-11

**Authors:** Roy Gommer, Roeland A. Bom, Thijs P. M. Fijen, Jan A. van Gils

**Affiliations:** 1 Department of Coastal Systems, NIOZ Royal Netherlands Institute for Sea Research and Utrecht University, Den Burg, Texel, The Netherlands; 2 Conservation Ecology Group, Groningen Institute for Evolutionary Life Sciences (GELIFES), University of Groningen, Groningen, The Netherlands; 3 Remote Sensing and GIS Center, Sultan Qaboos University, Al Khod, Oman; 4 Plant Ecology and Nature Conservation, Wageningen University & Research, Droevendaalsesteeg, Wageningen, The Netherlands; Hungarian Academy of Sciences, HUNGARY

## Abstract

Foragers whose energy intake rate is constrained by search and handling time should, according to the contingency model (CM), select prey items whose profitability exceeds or equals the forager’s long-term average energy intake rate. This rule does not apply when prey items are found and ingested at a higher rate than the digestive system can process them. According to the digestive rate model (DRM), foragers in such situations should prefer prey with the highest digestive quality, instead of the highest profitability. As the digestive system fills up, the limiting constraint switches from ingestion rate to digestion rate, and prey choice is expected to change accordingly for foragers making decisions over a relative short time window. We use these models to understand prey choice in crab plovers (*Dromas ardeola*), preying on either small burrowing crabs that are swallowed whole (high profitability, but potentially inducing a digestive constraint) or on larger swimming crabs that are opened to consume only the flesh (low profitability, but easier to digest). To parameterize the CM and DRM, we measured energy content, ballast mass and handling times for different sized prey, and the birds’ digestive capacity in three captive individuals. Subsequently, these birds were used in *ad libitum* experiments to test if they obeyed the rules of the CM or DRM. We found that crab plovers with an empty stomach mainly chose the most profitable prey, matching the CM. When stomach fullness increased, the birds switched their preference from the most profitable prey to the highest-quality prey, matching the predictions of the DRM. This shows that prey choice is context dependent, affected by the stomach fullness of an animal. Our results suggest that prey choice experiments should be carefully interpreted, especially under captive conditions as foragers often ‘fill up’ in the course of feeding trials.

## Introduction

Prey choice decisions in animals are thought to be the product of natural selection [1]. It is generally assumed that this has shaped carnivorous in such ways that they select prey that maximize their rate of energy gain [1] (but see some recent studies highlighting that predators also can make dietary decisions based on macro-nutritional composition or toxins [2–5]). This assumption was used in the optimal diet theory [6] to predict prey-choice decisions. The original and most frequently used optimal prey-selection model is the so-called ‘contingency model’ (CM) [1, 7]. CM predicts which prey items should be included in the diet based on their profitability. Each prey item *i* has a certain metabolizable energy content (*e*_*i*_) and a certain handling time (*h*_*i*_). Only prey items whose profitability (*e*_*i*_*/h*_*i*_) exceeds or equals long-term average energy intake rate should be included in the diet and consequently prey items with a lower profitability should be rejected.

The CM is supported by many empirical tests on for example birds, mammals and insects [1, 8, 9]. The CM applies to foragers which are so-called ‘handling-constrained’ [10], i.e. foragers that spend all their time searching and handling prey. Their energy intake is limited by the rate at which prey items can be found and handled. Problems with the CM arise when foragers are able to find and handle prey items faster than they can process them internally [11]. These foragers, instead of being handling-constrained, are ‘digestion-constrained’ [12–14]. Digestive pauses have to be taken before a new prey item can be ingested [15]. These digestive pauses cause a digestively constrained forager, obeying CM rules, not to maximise its long-term energy intake, because time to forage is lost during digestive pauses [14]. In this case, another optimal diet model should be considered.

The digestive rate model (DRM) [11, 16, 17] is an optimal diet model in which long-term intake rate is maximised under a digestive constraint. Tests of the DRM have first been restricted to herbivorous mammals [14, 18] and only relatively recently a few have been conducted on carnivorous birds [17, 19]. In this model, energy intake is limited by the rate of digestion and prey items are by and large selected on the basis of digestive quality (energy (*e*_*i*_) per unit of indigestible ballast mass (*k*_*i*_)), rather than profitability [19]. Foragers can use time, which would otherwise be lost to digestive pauses, to search for high quality (easy-to-digest) prey items [17]. Whether a forager needs to obey the CM or DRM thus depends on whether the forager is handling or digestively constrained.

Also the time horizon over which a forager wants to maximise its energy intake is important when considering optimal prey choice [19]. A forager aiming at maximising long-term energy intake should obey the rules of the DRM in case it faces, or is expected to face, a digestive constraint (i.e. has, or is expected to get, a full stomach). However, a forager aiming to maximise energy intake over a relatively short time interval [14], should obey the CM at the start of feeding when the stomach is still empty. As its stomach gradually fills up and the constraint switches from a handling to a digestive constraint, it should be optimal for a short-term rate maximizing forager to switch from CM-principles to DRM-principles [13, 20, 21].

Here we will use both diet models to understand prey choice decisions in crab plovers (*Dromas ardeola*), a tropical shorebird that primarily consumes crabs, but also consumes fish and benthic invertebrates [22]. In our study area in the Sultanate of Oman, crab plovers mainly prey on two types of crabs: small burrowing crabs, *Macrophthalmus sulcatus* (hereafter *Macrophthalmus*), that are ingested whole and potentially induce a digestive constraint or large swimming crabs, *Portunus segnis* (hereafter *Portunus*) that are opened to consume the flesh only, potentially inducing a handling constraint. *Portunus* is opened, since it is physically impossible to swallow the whole crab. The processing dichotomy between these two species makes the system ideal to study prey choice in the light of the CM and DRM. We tested, under captive conditions, the prey choice of crab plovers when offered small *Macrophthalmus*, small *Portunus* and large *Portunus*. Both a dichotomous prey choice experiment (empty stomach) as well as a cafeteria experiment have been performed to test for changes in prey choice as the stomach fills up. We parameterized both the CM and DRM by estimating the energy content of the crabs, the ballast mass of the crabs and the handling times of crab plovers on different crabs. The predictions of the CM and DRM were used to explain the outcomes of our prey choice experiments.

## Methods

### Study area & study species

The study was conducted on the relatively pristine mudflats of Barr al Hikman peninsula, located at the central-east coast of the Sultanate of Oman (20.6° N, 58.4° E). Barr al Hikman is one of the largest and most important wetland areas in the Middle East and supports large numbers of shorebirds [23]. Among them is the crab plover (*Dromas ardeola*), our study species. About 8,000 of these conspicuous black-and-white birds winter in the area, making it the most important wintering area for this species [24]. Its breeding range covers the north-western Indian Ocean and the Red Sea, while its wintering range covers most of the Indian Ocean [25, 26]. Throughout its wintering range the diet of crab plovers mainly consists of crabs, but other invertebrates and fish are also eaten [27, 28].

### Captive birds

The birds to be held in captivity were caught during the night using mistnets, early November 2015. After capture, these birds were housed in an aviary (2.5 m width x 2.5 m length x 1.25 m height), made out of wood and nets. It took about a week for them to get accustomed to these new conditions. During this start-up phase they were fed a mixed diet of both crab species to be used in our experiments in order to prevent them from getting used to a single prey species. After catching the weight of the birds initially decreased, but stabilized after about a week at on average 79% (SD ± 4%) of the catching weight. Two birds suffered from leg cramp, presumably caused by stressful conditions of catching, from which one recovered during the week before the experiments, leaving us with three birds to be used in our experiments. After this week, each bird was assigned to a series of feeding trials. To be able to parameterize the CM, we measured handling time in relation to crab size. To be able to parameterize the DRM, we conducted a maximum intake experiment. Prey choice was tested in a dichotomous prey choice experiment and a cafeteria experiment. The birds were released by the end of November 2015.

## Animal welfare note

All work was performed under full permission by the authorities of the Ministry of Environment and Climate Affairs (MECA), Sultanate of Oman under permit number 31/2015. No animal experimentation ethics guidelines exist in The Sultanate of Oman. However, the experiment was carried out in strict accordance with Dutch animal experimentation guidelines. The NIOZ Royal Netherlands Institute for Sea Research has been licensed by the Dutch Ministry of Health to perform animal experiments under license number 80200. This license involves capture and handling of animals, and performing experiments, which nonetheless should be individually approved by the Animal Experimentation Committee (DEC) of the Royal Netherlands Academy of Arts and Sciences (KNAW). The DEC does not provide permits for experiments in foreign countries, but provided approval for equivalent experiments in the Netherlands by the same persons under permit number NIOZ 10.05, involving the capture of birds, performing non-invasive experiments consisting of prolonged diets of natural food types (i.e. foods that regularly occur in the diet of wild birds) and repeated gizzard size measurements, and includes permission to release healthy animals in the wild after the experiment. All possible efforts were made to minimize physical and mental impact on the experimental animals. Each bird was weighed and visually inspected for general condition daily. The reasons for the experiment to take place in the Sultanate of Oman were purely scientific and by no means to avoid ethics guidelines. All experimental animals were released in the wild in healthy condition after the experiment.

### Prey species

For all experiments, we used *Macrophthalmus* and *Portunus*. As profitability and digestive quality of *Portunus* was expected to scale substantially with size, we used two size classes of these species: a small (carapax width: 30–50 mm) and a large (carapax width: 60–90 mm). For the interest of this study, we report for both crab species the metabolizable energy content, the undigestible (inorganic) part and the total mass (undigestible + digestible). Following Zwarts and Wanink (29) we used ash-free dry mass (AFDM) as our measure of metabolizable energy, or digestible part of the prey. It is reasonable to assume that the energy value per unit AFDM does not vary with species and size [29]. Likewise, the ash content of the prey was used as the undigestible part of the prey. The dry mass (DM) of the prey was used as the total mass, which was defined as the undigestible + digestible part of the prey. To predict for each crab offered in the experiments its AFDM, its ash content and DM on the basis of its size, we fitted regression models relating crab size to AFDM, ash content and DM for individuals of both crab species, collected in November 2015 and covering the entire size range found in the field. Collected crabs were stored in formalin and transported to the NIOZ Royal Netherlands Institute for Sea Research. Here, the width of each crab was measured to the nearest mm. Next, crabs were dried for three days at 55–60 °C in a ventilated oven, after which DM was obtained to the nearest 0.01 g. Subsequently the crabs were incinerated at 550 °C for two hours and the ash mass was obtained. AFDM was calculated as the DM minus the ash mass. Non-linear regression models (power function: y = ax^b^; Table 1) were fitted using R-package gnls [30]. Crab plovers do not eat the carapaxes of large *Portunus*. Depredated carapaxes were collected and their DM, AFDM and ash was determined using the same methodology as mentioned above. Regression models (Table 1) relating crab width to empty carapaxes were made in the same way as the other regression models and were subtracted from the previous mentioned regression models to determine the true ingested flesh by crab plovers. We assume that the energy loss due to the formalin fixation is similar across species and size classes [29, 31].

**Table 1:** AFDM (mg) versus crab width (mm), ash mass (mg) versus crab width (mm), DM (mg) versus crab width (mm) and handling time versus crab width (mm) for both crab species. For *Portunus* we also determined the carapax AFDM (mg), ash mass (mg) and DM (mg) versus crab width (mm).

model	*Macrophthalmus*	*Portunus*
AFDM ~ Size	*y* = 4.05e-02*x*^2.76^	*y* = 4.20e-02*x*^2.53^
Carapax AFDM ~ Size	-	*y* = 8.89e-02*x*^1.97^
Ash ~ Size	*y* = 3.66e-02*x*^2.78^	*y* = 1.90e-02*x*^2.65^
Carapax ash ~ Size	-	*y* = 1.58e-02*x*^2.58^
DM ~ Size	*y* = 7.19e-02*x*^2.80^	*y* = 5.96e-02*x*^2.58^
Carapax DM ~ Size	-	*y* = 4.13e-02*x*^2.44^
Handling ~ Size	*y* = 0.19*x*^0.91^	*y* = 0.003*x*^2.72^

### CM

In order to make predictions based on the CM, we calculated the profitability (*e*_*i*_/*h*_*i*_) of the prey in a series of feeding trials in which all three birds were offered differently sized prey items. We used prey items over the entire size range found in the field. Feeding trials were conducted during the morning to make sure birds had an empty stomach, so that they had the same motivation to eat. Furthermore, feeding trials were conducted on single birds to make sure that interference did not affect our results. All trial were filmed (Canon VIXIA HG21). To establish the profitability (*e*_*i*_/*h*_*i*_) of crabs, we first calculated the energy content (*e*_*i*_) using the AFDM of each prey item offered, calculated by using the equations in Table 1. The handling time (*h*_*i*_) was measured from the moment of attacking the prey till the moment of swallowing the prey. Pauses during handling were excluded from the handling time. We analyzed the video’s using ‘The Observer’ package (v. 5.0, Noldus Information Technology). Profitability was then calculated by dividing AFDM (*e*_*i*_) by handling time (*h*_*i*_). Linear mixed-effect models with crab width against profitability were fitted to test for a relation between profitability and crab width. We used crab width as a fixed effect and bird as a random effect. To compare the profitability between prey species we also used a linear mixed-effect model with crab species as a fixed effect and bird as a random effect. To fit the profitability versus size curves we used power functions: (y = ax^b^), using R-package gnls [30].

### DRM

To make predictions based on the DRM, we first experimentally determined whether ash (undigestible part of the prey), AFDM (digestible part of the prey) or DM (undigestible + digestible part of the prey) is the ballast mass that sets a digestive constraint in crab plovers, following the same procedure as van Gils et al (15). We assumed that the rate at which digestively constrained crab plovers can process the ballast mass of a prey will be constant across prey types [17]. This means that if the ballast mass of a prey item is double compared to the ballast mass of another prey item, the long term numerical intake rate on the prey item with the high ballast mass will be twice as low as the long term numerical intake rate on the prey item with the low ballast mass [15]. The rate at which prey can be consumed is given by the formula: $y=\frac{1}{x}c$ (where *y* is numerical intake rate (IR); *x* is DM, AFDM or ash content of the prey; and *c* is digestive constraint) [15].

#### Maximum-intake experiment

To determine the digestive constraint of crab plovers we offered the captive birds *ad libitum* food, being either *Macrophthalmus*, small sized *Portunus* or large sized *Portunus*. Each feeding trial lasted two hours and was repeated once, so we conducted (3 birds × 3 diets × 2 repetitions) 18 feeding trials in total. Three feeding trials were excluded because of camera failure. Trials were filmed (GoPro4) and intake was scored using ‘The Observer’ package (v. 5.0, Noldus Information Technology). Cumulative intake (# prey items) was plotted versus time (minutes) to estimate the long-term intake rate (slope). We estimated long-term intake rate (IR) using the slope between the point of first saturation (last crab ingestion before first digestive break) and the end point (last crab ingestion observed) of a feeding trial [32, 33]. The first saturation point was the point where crab plovers had not eaten for more than seven minutes which we interpreted as a digestive pause. We also inspected this graphically to confirm that the starting point was correct. IR of all the trials was then plotted versus average DM, AFDM and ash content of the crabs that were eaten during the experiment. A line was fitted using a linear mixed-effect model on log-transformed data with bird as a random effect. We tested whether the slope of this model differed significantly from -1, because a slope of -1 implies that there is a fixed amount of ballast mass, coined *c*, a stomach can process per unit of time [15]. This follows mathematically when log-transforming the formula: $y=\frac{1}{x}c$. We did this for DM, AFDM and ash content to determine what constrains the food intake of crab plovers (the one that does not differ from -1).

#### The digestive rate model

To parameterize the digestive rate model (DRM) we used the prey characteristics of both prey species. We plotted profitability (*e*_*i*_/*h*_*i*_) of both species versus ballast intake (*k*_*i*_/*h*_*i*_) [17]. In addition, we plotted the digestive constraint. For *k*_*i*_ we used ash content (g), because that is what constrains the food intake of crab plovers (see Results).

### Dichotomous prey choice experiment

**Fig 1:**
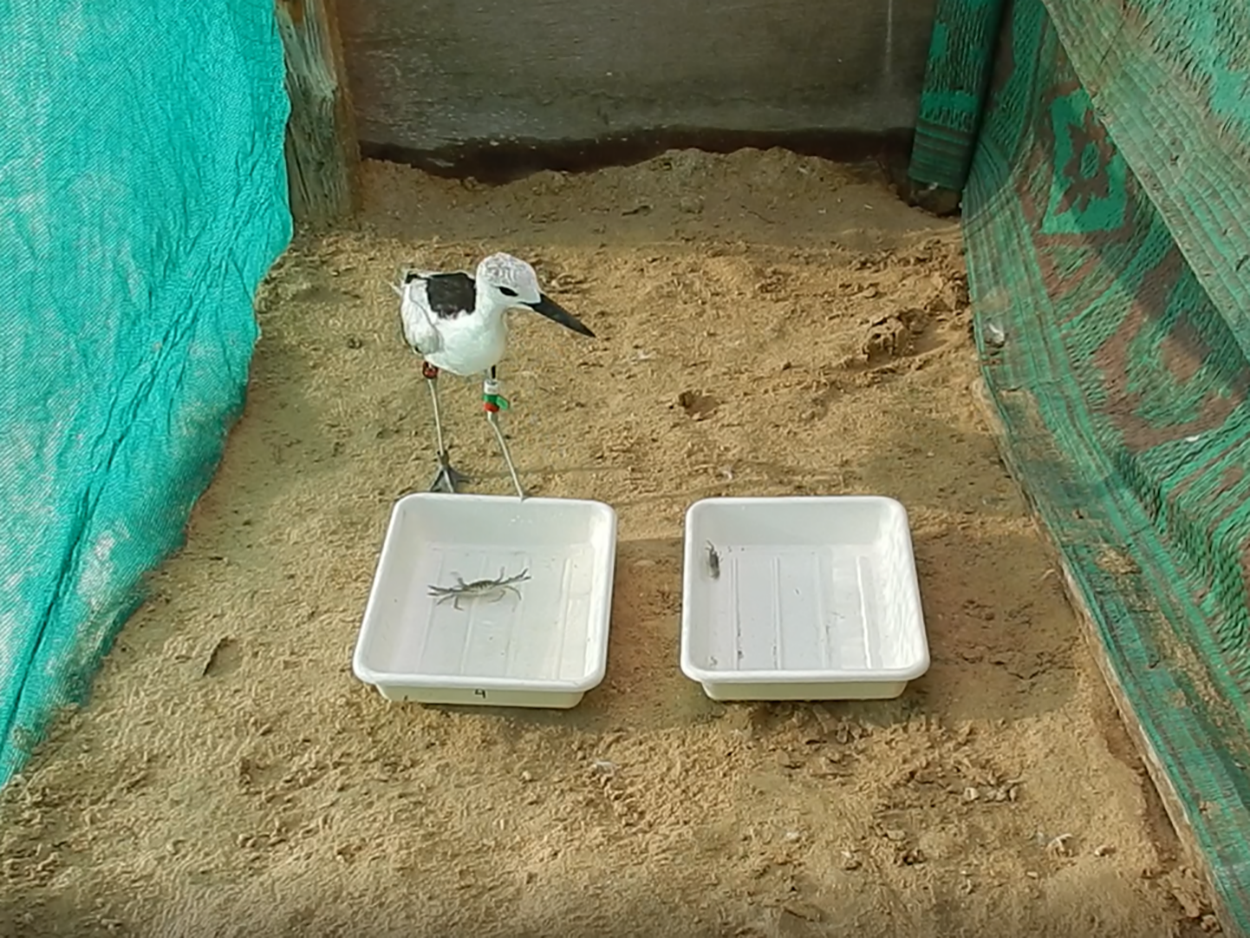
Crab plover facing two different prey items in two separated trays. The left tray contains a small *Portunus* and the right tray a *Macrophthalmus*.

Crab plovers were offered two different prey items in two separated trays (Fig 1). Prey species were randomly assigned to different sides (left/right). Crab plovers were brought into the experimental aviary on the opposite side of the trays to make sure they could see both prey items when walking towards the trays before making a choice. We conducted several trials per bird, but all on different days. Trials were conducted during the morning when birds had not eaten for the whole night to make sure their stomach was empty. We offered each bird three combinations: *Macrophthalmus* versus small *Portunus* (18 trials), *Macrophthalmus* versus large *Portunus* (17 trials) and small *Portunus* versus large *Portunus* (18 trials). For crab characteristics of the crabs offered see Table 2. To test prey preference, we used the dichotomous prey test [34]. We used a generalized linear model with prey choice as our response variable and the different prey types as our predictor variables. A quasibinomial model was used and the cardinal preference rank was calculated for each prey type. The cardinal preference rank of large *Portunus* was set to zero (no SE) as we compared *Macrophthalmus* and small *Portunus* to large *Portunus*.

**Table 2:** Crab characteristic of the crabs offered in the dichotomous prey choice experiment. The number of crabs offered (n) as well as the average crab size (± SD) is shown. Average (± SD) AFDM (mg), handling time (s) and ash (mg) was calculated based on the crab sizes of each individual crab using the formulas in Table 1. Average (± SD) profitability (*e*_*i*_/*h*_*i*_) was calculated by dividing AFDM (mg) by handling time (s) for each individual crab. Average (± SD) digestive quality (*e*_*i*_/*k*_*i*_) was calculated by dividing AFDM (mg) by ash (mg) for each individual crab.

	n	Size (mm)	AFDM (mg)	Handling time (s)	Profitability (*e*_*i*_/*h*_*i*_)	Ash (mg)	Digestive quality (*e*_*i*_/*k*_*i*_)
*Macrophthalmus*	35	19.1 ± 2.4	145 ± 51	2.8 ± 0.3	50.75 ± 11.94	139 ± 50	1.04 ± 0.00
small *Portunus*	36	42.6 ± 4.0	420 ± 108	82.6 ± 21.2	5.08 ± 0.00	145 ± 38	2.90 ± 0.01
large *Portunus*	35	68.4 ± 5.2	1490 ± 314	297.1 ± 63.9	5.03 ± 0.02	534 ± 117	2.80 ± 0.02

### Cafeteria experiment

Because prey choice might differ depending on the internal state (fullness of the stomach) of the crab plovers, we offered them *ad libitum* food of all three prey types, i.e. *Macrophthalmus* (on average 17 crabs), small *Portunus* (on average 9 crabs) and large *Portunus* (4 crabs), with each prey type in a separated tray. For crab characteristic of the crabs offered see Table 3. Each feeding trial lasted approximately two hours and was filmed to determine the exact moments of ingestion in time (GoPro4). From these videos the cumulative numeric intake was scored using ‘The Observer’ package (v. 5.0, Noldus Information Technology). We also scored which prey type was ingested. After each feeding trial we counted the crabs that were left to calculate the number of crabs the crab plover had eaten. Two of the three birds were used (the third was not used because of time limitation) on which we both conducted two feeding trials, so we had four trials in total. Trials were conducted on four different days and for each bird there was a day in between each trial. Birds that entered the trials had not eaten for at least four hours to make sure their stomach was empty. For the purpose of this study, two trials could not be used because in trial 3 the crab plover had eaten all *Macropthalmus* before reaching its digestive constraint and in trial 4 the crab plover stopped eating after the camera failed. This left us with two successful trials on two different birds. Trial 2 suffered from unfortunate camera failure after 15 minutes. Within this time period the experimental bird had reached its digestive constraint, and by counting the crabs that were left at the end of the trial we could calculate the number of crabs that were eaten after the camera failed. These crabs were included in the results but we do not know when these crabs were eaten and in which order.

**Table 3:** Crab characteristic of the crabs offered in the cafetaria experiment (2 trials). The number of crabs offered (n) as well as the average crab size (± SD) is shown. Average (± SD) AFDM (mg), handling time (s) and ash (mg) was calculated based on the crab sizes of each individual crab using the formulas in Table 1. Average (± SD) profitability (*e*_*i*_/*h*_*i*_) was calculated by dividing AFDM (mg) by handling time (s) for each individual crab. Average (± SD) digestive quality (*e*_*i*_/*k*_*i*_) was calculated by dividing AFDM (mg) by ash (mg) for each individual crab.

	n	Size (mm)	AFDM (mg)	Handling time (s)	Profitability (*e*_*i*_/*h*_*i*_)	Ash (mg)	Digestive quality (*e*_*i*_/*k*_*i*_)
*Macrophthalmus*	34	20.0 ± 2.3	162 ± 47	2.9 ± 0.3	54.79 ± 11.17	156 ± 46	1.04 ± 0.00
small *Portunus*	18	44.3 ± 4.3	470 ± 117	92.5 ± 22.9	5.08 ± 0.00	163 ± 41	2.89 ± 0.01
large *Portunus*	8	73.4 ± 5.5	1803 ± 357	360.4 ± 72.8	5.01 ± 0.02	650 ± 133	2.78 ± 0.02

## Results

### Feeding behaviour

As anticipated, the crab plovers swallowed the *Macrophthalmus* always whole, while *Portunus* was always stripped from the carapax, legs and pincers, and only the flesh was eaten.

### CM

**Fig 2:**
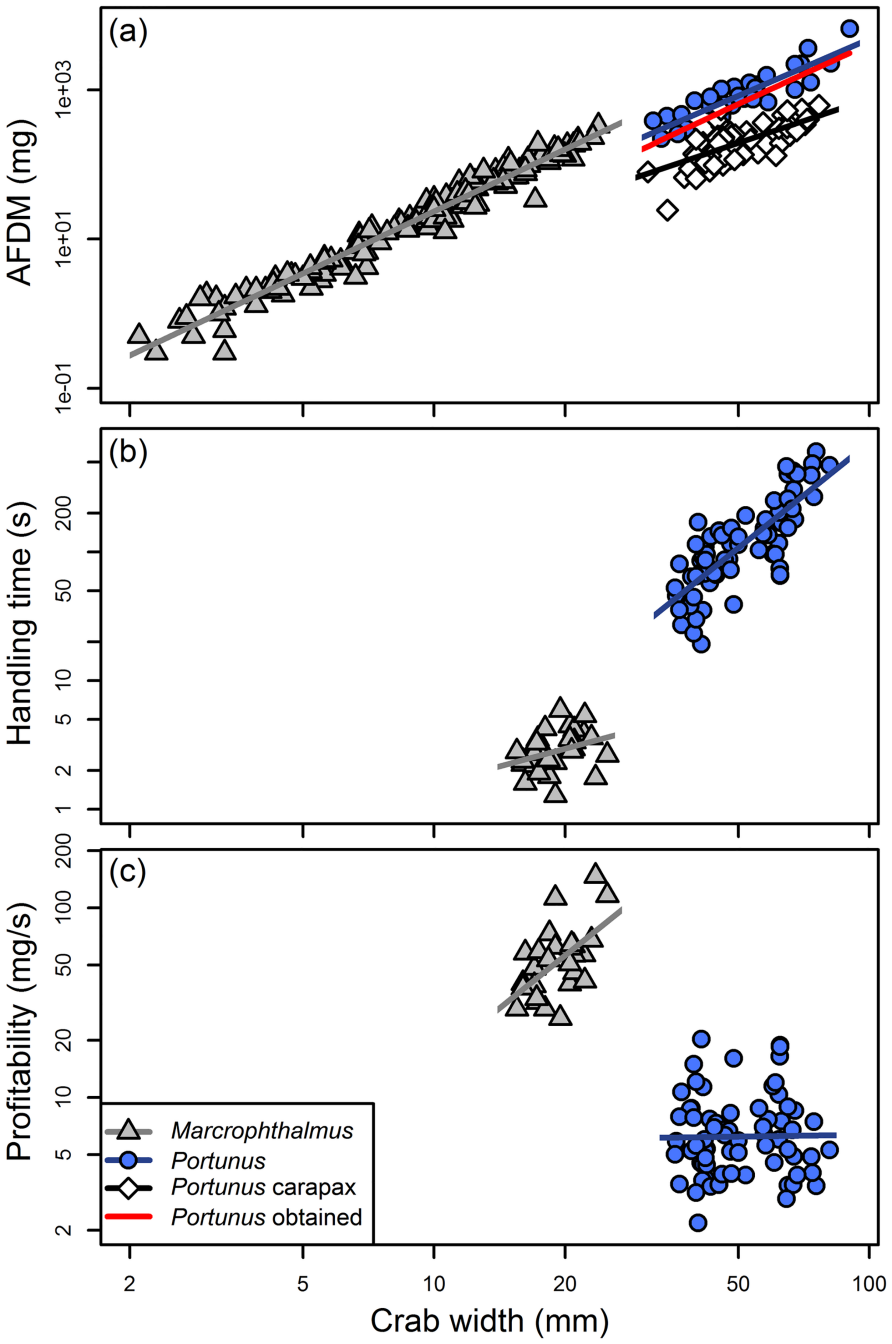
(a) AFDM (*e*_*i*_) plotted versus crab width (mm) (note the logarithmic axes). Grey triangles represent *Macrophthalmus* and blue dots represents *Portunus*. Non-filled rhombs represent the amount of AFDM (*e*_*i*_) that was left in depredated carapaxes of *Portunus*. The amount left in the carapaxes in terms of AFDM (*e*_*i*_) was subtracted from the AFDM (*e*_*i*_) of intact *Portunus*, yielding the amount of AFDM (*e*_*i*_) obtained by crab plovers, superimposed with a red line. For formulas see Table 1. (b) Handling time (h_*i*_) plotted versus crab width (mm) (note the logarithmic axes). Handling time does not increase significantly with size for *Macrophthalmus* (superimposed with a grey line), while for *Portunus* handling time significantly increases with size (superimposed with a blue line). For formulas see Table 1. (c) Profitability (AFDM (*e*_*i*_) / handling time (*h*_*i*_)) plotted versus crab width (mm) (note the logarithmic axes). Grey triangles represent *Macrophthalmus* and blue dots represent *Portunus*. Profitability significantly increases with size for *Macrophthalmus* (*y* = 0.22*x*^1.85^; superimposed with a grey line), while for *Portunus* profitability does not significantly increase with size (*y* = 5.46*x*^0.03^; superimposed with a blue line).

*Macrophthalmus* had a higher profitability than *Portunus* (df = 96, t-value = -14.81, p < 0.001; Fig 2c) which was mainly caused by the short handling times on *Macrophthalmus*. Handling times were much larger for *Portunus* ranging from 50 to 500 seconds versus 2 to 5 seconds for *Macrophthalmus* (Fig 2b). So following the CM crab plover should always choose the more profitable *Macrophthalmus*. We found a positive exponential relation between profitability and crab size in *Macrophthalmus* (df = 24, t-value = 3.59, p = 0.002). Crab size did not affect profitability in *Portunus* (df = 68, t-value = 0.13, p = 0.897).

### DRM

#### Maximum intake experiment

**Fig 3:**
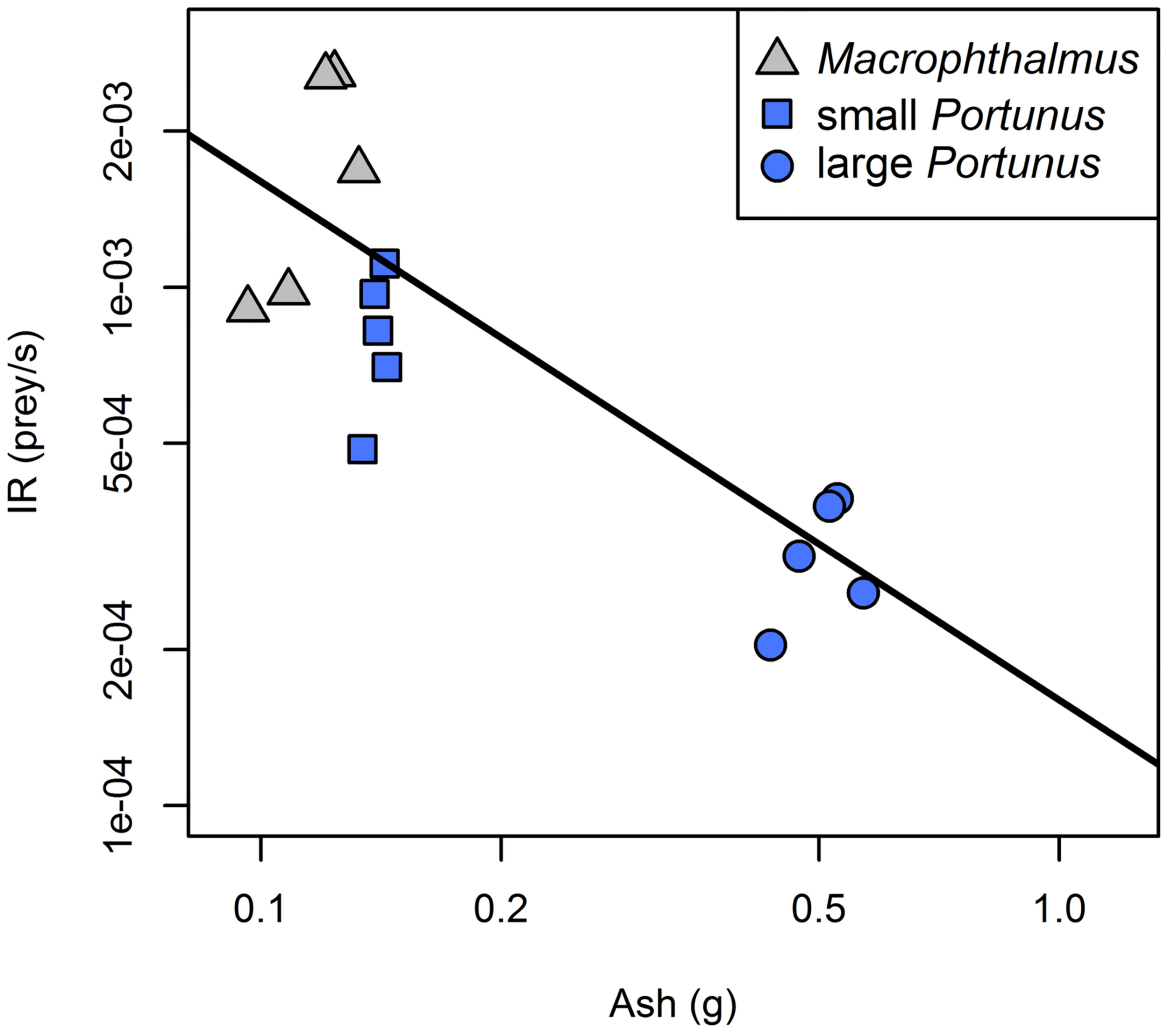
Intake rate (prey/s) plotted versus ash content of that prey (g/prey) in the *ad libitum* experiment. The line represents the relation: ^10^log(IR) = -3.80 – ^10^log(ash).

The slope of the relationship between the log-transformed IR and the different ballast weights was not significantly different from -1 for ash (slope = -0.94, p = 0.737; Fig 3), marginally significantly different from -1 for DM (slope = -0.75, p = 0.053) and significantly different from -1 for AFDM (slope = -0.64, p = 0.004). This means that the variation in numerical intake rate between prey items can best be explained by the ash content of the prey: i.e. if a prey item contains twice as much ash compared to another prey item, the numerical intake rate on the prey item with the high ash content will be twice as low as the numerical intake rate on the prey item with the low ash content. Therefore, ash content (*k*_*i*_*)* appears to constrain the long-term intake rate of crab plovers. Using the intercept of the obtained relationship (^10^log(IR) = -3.80–0.94 × ^10^log(ash)), we found crab plovers to have a digestive constraint of (10^−3.80^) 0.16 mg of ash per second.

#### The digestive rate model

**Fig 4:**
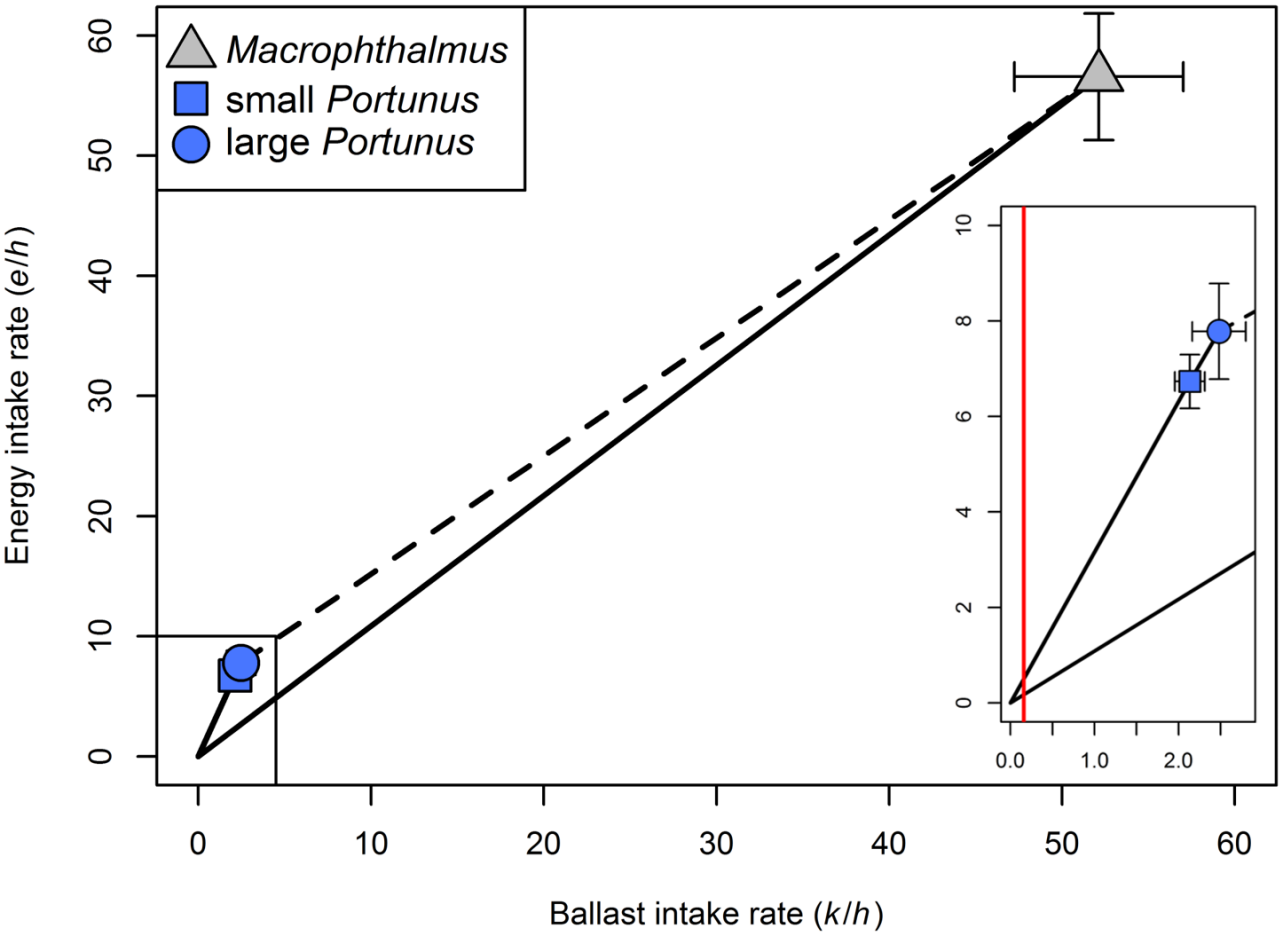
The digestive rate model. Energy intake rate (*e*_*i*_/*h*_*i*_) (mg/s) was plotted versus ballast (ash) intake rate (*k*_*i*_/*h*_*i*_) (mg/s) for both crab species. The grey triangle represents *Macrophthalmus* (n = 28), the light blue square represents small *Portunus* (n = 23) and the blue dot represents large *Portunus* (n = 43). Arrows represent the standard error of the mean. The inset gives a more detailed view of the *Portunus* size classes. The long solid black line represents the slope in terms of energy per ballast (*e*_*i*_/*k*_*i*_) for *Macrophthalmus*, whereas the short solid black line on the left represents the slope for *Portunus*. The solid red line represents the digestive constraint (*c*) (0.16 mg/s ash). For both prey species *k*_*i*_/*h*_*i*_ > *c*, which means that the highest long-term energy gain can be obtained by choosing the prey with the highest slope (in this case *Portunus*).

While *Macrophthalmus* had a higher profitability than *Portunus*, this was the other way around for digestive quality (slope (*e*_*i*_/*k*_*i*_)). We found the digestive constraint (*c*) to be on the left side of graph (see inset Fig 4), which means that *k*_*i*_/*h*_*i*_ > *c* for all prey types. Thus, crab plovers that face a digestive constraint should always choose the better digestible *Portunus*.

### Dichotomous prey choice experiment

**Fig 5:**
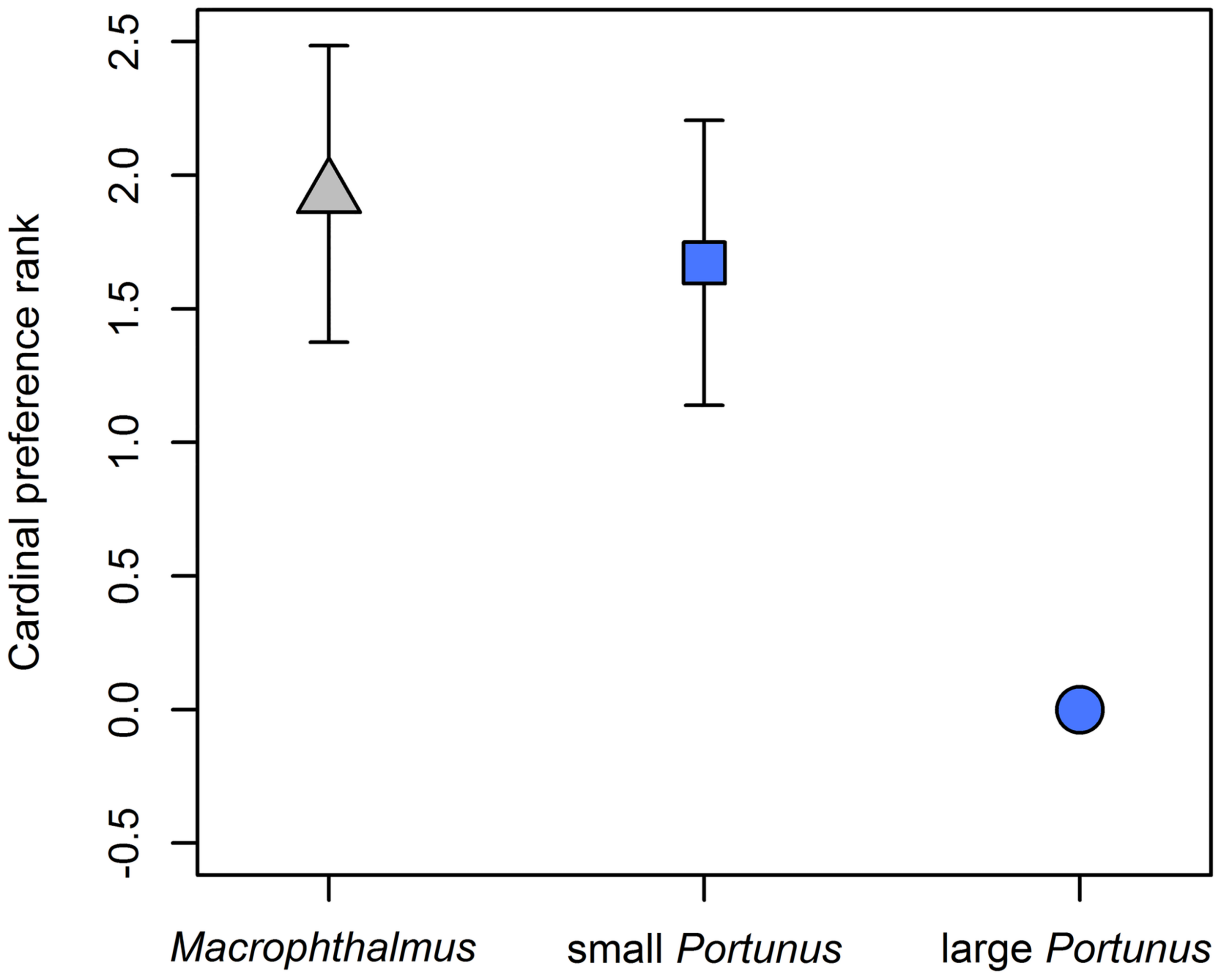
Dichotomous prey choice experiment. The cardinal preference rank is plotted against prey type. A higher cardinal preference rank (*y*-axis) indicates a higher preference over the other prey species. Arrows represent the standard error of the mean. Large *Portunus* is set to zero (no SE) as we compared *Macrophthalmus* and small *Portunus* to large *Portunus*. We found crab plovers to prefer *Macrophthalmus* over large *Portunus* (t-value = 3.480, p = 0.001). We also found crab plovers to prefer small *Portunus* over large *Portunus* (t-value = 3.135, p = 0.003). We found no difference in preference for *Macrophthalmus* versus small *Portunus* (t-value = -0.587, p = 0.560).

*Macrophthalmus* was preferred over large *Portunus* (t-value = 3.480, p = 0.001; Fig 5). Also small *Portunus* was preferred over large *Portunus* (t-value = 3.135, p = 0.003; Fig 5). We found no difference in preference between *Macrophthalmus* and small *Portunus* (t-value = -0.587, p = 0.560; Fig 5).

### Cafeteria experiment

**Fig 6:**
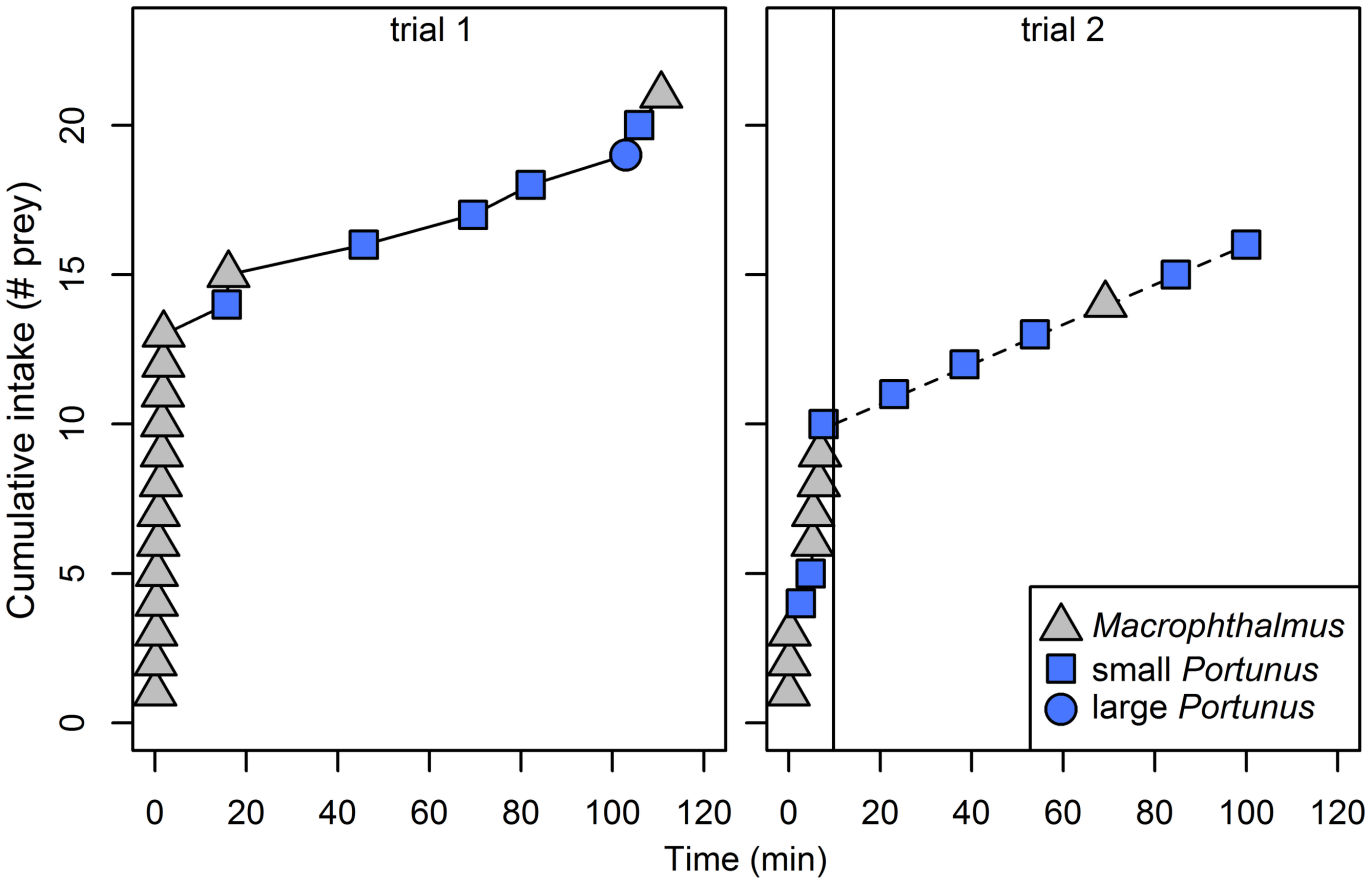
Cafeteria experiment. Cumulative intake (# prey) has been plotted on the *y*-axis and time (min) on the *x*-axis. Each point represents a crab that has been eaten. For trial 1 we obtained the whole video. For trial 2 the camera failed after some time. The vertical line represents the moment of camera failure. We know the number of crabs that were eaten after camera failure based on the number of crabs that were left after the feeding trials. These crabs have been plotted on the right side of the vertical line. Note that we do not know when these crabs were eaten and in which order. For simplicity, we plotted them in constant intervals to the end of the feeding trial. The birds had an initial preference for *Macrophthalmus*. In both trials the crab plover switched its prey choice from *Macrophthalmus* to small *Portunus* to the end of the feeding trial. The trials were conducted on two different birds.

In both feeding trials, there was an initial preference for *Macropthalumus*, i.e. in both feeding trials the crab plovers started eating a number of *Macrophthalmus*. The preference switched to *Portunus* in the course of the feeding trial after crab plovers had reached their digestive constraint (two out of two; Fig 6). In the two trials that did not succeed we also observed the initial preference to be *Macrophthalmus*.

## Discussion

We found crab plovers to switch their prey preference depending on their stomach fullness. When offering crab plovers all prey types in *ad libitum* quantities, crab plovers switched their preference from the highly profitable *Macrophthalmus* to the high-quality *Portunus* after their stomach filled up to full capacity, which we assumed to be indicated by the observed breaks (Fig 6). This suggests that crab plovers integrate their decisions over a relatively short time window. Hence, on an empty stomach they obeyed the CM, while they obeyed the DRM with a full stomach.

In addition, we also found that prey choice depends on the expected future prey items. When crab plovers with an empty stomach were offered two prey items only, they preferred *Macrophthalmus* over large *Portunus* (Fig 5), which is according to the CM. However, when offering them *Macrophthalmus* and small *Portunus*, we did not find a preference for the more profitable *Macrophthalmus* (Fig 5) which is against the predictions of the CM. This result differs from our cafeteria experiment, where we found that crab plovers with an empty stomach always choose *Macrophthalmus*. This discrepancy might be explained by the fact that in our dichotomous choice experiment we only offered two prey items. Crab plovers did not know what was coming after these two preys and might decide to take the one that yields the most energy first in spite of a longer handling time, i.e. the small *Portunus*, to minimize the risk of starvation [35, 36]. This suggests that crab plovers anticipate future energy gains within a certain time-horizon and this might aid in shaping prey choice decisions.

Alternatively, the importance of the nutritional and toxic composition of the prey species might play a role. Stephens and Krebs (1) assumed that the diet of carnivorous animals mainly consists of prey with approximately the right balance of nutrients and that carnivorous animals make dietary decisions solely based on energy content, but recent studies showed some vertebrate and invertebrate predators to make dietary decisions based on macro-nutritional composition, rather than energy content [2, 4, 5]. Also the presence of toxins in certain prey types can affect prey choice decisions of foragers [3]. The observed switch in prey choice could thus potentially also be explained by foragers aiming at achieving nutritional targets, or foragers being limited by toxic constraints. However, given that maximum intake rates in terms of ash were equal for both prey species, we don’t expect one of our prey species to be toxic [37]. Furthermore, studies showing that carnivores balance their diet based on nutrients do not report sudden shifts, as we observed in crab plovers, but rather show a balanced mixed diet [38, 39] or a switch over relatively long time periods, i.e. days or seasons, for instance to prepare for breeding [21]. We thus believe that the observed diet switch in crab plovers is primarily driven by energy and shaped by stomach fullness. That crab plovers may encounter digestive problems can be expected as 47% (SD ± 8%) of *Macrophthalmus* consists of inorganic mass.

It is important to note that prey choice in the field may differ from our results, as conditions in the field differ from the conditions in our experiment. Problems in testing optimal prey choice in the field may arise because these models often fail when using mobile prey items, for example due to escape behaviour of prey [9]. In our experiment, both species were readily available (same densities) and catchable (search time = 0), but this is certainly not true in the field where *Macrophthalmus* are known to escape into their burrows when a predator is near, which may be a much more effective escape behaviour than hiding in the sand near the surface like *Portunus* do. This may negatively affect searching efficiency on *Macrophthalmus*, which in turn potentially affects prey choice, especially when crab densities are low and/or when searching for *Macrophthalmus* and searching for *Portunus* are mutually exclusive. Furthermore, prey choice could differ in case crab plovers in the field are not energy maximisers, as assumed here, but instead are time minimisers [40]. I.e. if crab plovers aim to minimise time foraging (searching and handling) and take digestion for granted, we could expect that crab plovers should again switch to the more profitable prey, i.e. *Macrophthalmus*. In our experiment, the birds had lost weight during the pre-experimental period which might have turned them into energy maximisers in order to recover. Finally, in the field the optimal prey choice might also be affected by the interaction with the social environment, with other crab plovers foraging on crabs [41]. This can result in crab plovers preferring prey items with short handling times, i.e. *Macrophthalmus*, in order to minimize the chance for kleptoparasitism. It could also influence the searching time on *Macrophthalmus*, as the presence of a lot of crab plovers might make them escape into their burrows and as a result makes *Macrophthalmus* a less attractive prey. Detailed observations should give insight in which strategy is adopted by crab plovers in the field.

In conclusion, we show that under captive conditions, when crab plovers are in handling constrained circumstances, the CM predicts their prey choice well when offering *ad libitum* prey (initial phase in Fig 6). However, when offering only two prey items, the CM only partially predicts prey choice, as time-horizon and anticipation effects come into play. When crab plovers become digestively constrained, the prey choice decisions are in line with the DRM (end phase in Fig 6). Our results indicate that prey choice is not necessarily dependent on the CM (handling constraint) or the DRM (digestive constraint) alone, but is context dependent in terms of stomach fullness. This follows the predictions of Whelan and Brown (20) stating that food choice is dynamic and depends on an animal’s digestive state. Based on our results it could be expected that stomach fullness is an important parameter for understanding prey choice. This has been shown in several experiments when offering differently sized prey items of the same species [42, 43]. Yet we could only find one study with experimental data [21] to substantiate, and one with field data [44] to suggest a switch of prey species based on stomach fullness as we found here. Thus, the generalization of how stomach content effects prey choice needs to be further studied.

That the stomach fullness affects prey choice might have serious implications when conducting prey choice experiments in captivity. Several laboratory studies have tested optimal diet theory on foragers having an empty stomach [17, 45, 46] or do not mention the context (i.e. stomach fullness) under which prey choice was tested [47, 48]. Optimal diet theory has sometimes failed [9], which, as we argue, could result from not taking into account the stomach fullness of a forager. Thus, precaution in terms of (changes in) stomach fullness should be taken when conducting lab experiments on prey choice decisions.
